# Laparoscopic Natural Orifice Specimen Extraction Surgery versus Conventional Surgery in Colorectal Cancer: A Meta-Analysis of Randomized Controlled Trials

**DOI:** 10.1155/2022/6661651

**Published:** 2022-01-18

**Authors:** Zhuqing Zhou, Lin Chen, Jie Liu, Fang Ji, Yuanyuan Shang, Xudong Yang, Yao Yang, Chuangang Fu

**Affiliations:** Department of Colorectal Surgery, Department of General Surgery, Shanghai East Hospital, Tongji University School of Medicine, 150 Jimo Road, Shanghai 200120, China

## Abstract

**Objective:**

This study was to quantitatively synthesize data in randomized controlled trials (RCTs) of laparoscopic resection comparing natural orifice specimen extraction (NOSE) versus conventional laparoscopy (CL) in colorectal cancer.

**Methods:**

We identified eligible RCTs by searching seven electronic databases (PubMed, Cochrane Library, Embase, Web of Science, CNKI, CQVIP, Wanfang, and Sinomed). Mean differences (MDs) between groups with 95% confidence intervals (CIs) were used for continuous outcomes. Event rate ratios (RRs) were also calculated with their 95% CIs.

**Results:**

1,569 citations were identified from electronic database as of June 2020, and finally, 21 RCTs involving 2,112 patients met the study eligibility criteria and were included. Compared to the CL group, NOSE had longer operation time (MD: 8.14 min, 95% CI: 3.02 to 13.25, and *p* < 0.01), less estimated blood loss (-10.64 ml, 95% CI: -14.92 to -6.36, and *p* < 0.01), less hospital stay after surgery (-2.21 days, 95% CI: -3.36 to -1.06, and *p* < 0.01), shorter time of gas passage after surgery (-0.58 days, 95% CI: -0.82 to -0.34, and *p* < 0.01), better pain score (-1.06, 95% CI: -3.74 to -0.37, and *p* < 0.01), and improved cosmetic scores (1.93, 95% CI: 0.77 to 3.10, *p* < 0.01). Rate ratios of total complications, infection, and incision infection all favored NOSE surgery, with RRs (95% CIs) of 0.81 (0.71 to 0.93), 0.34 (0.21 to 0.54), and 0.24 (0.12 to 0.51), respectively.

**Conclusion:**

This report appeared the first comprehensive meta-analysis of RCTs to synthesize data of laparoscopic resection with NOSE versus conventional laparoscopy. NOSE surgery seemed favorable with shorter hospital stay, less pain score, a shorter time to recover along with better cosmetic scores, and less postoperative complications.

## 1. Introduction

Colorectal cancer (CRC) remains one of primary causes of cancer-related morbidity and mortality worldwide [1]. As one of the treatment options, laparoscopic surgery has been accepted for decades widely [2]. In recent years, natural orifice specimen extraction surgery (NOSES) is gradually practicing in CRC's treatment and hence causes widespread interests among surgeons [3]. It is reported that NOSE surgery would reduce access trauma in laparoscopic colorectal surgery, with alleviated postoperative pain, faster patient recovery, and a favorable long-term outcome regarding cosmesis and incisional hernia rate [4]. However, a NOSE surgery guideline with adequate evidence has not been formulated to date yet. There were also negative arguments that NOSE surgery may be a risk factor of bacterial contamination of the peritoneal cavity [5]. Nevertheless, relevant studies on NOSE are increasing year by year while few meta-analyses, especially of randomized controlled trials (RCTs), have been carried out. As a result, this topic is still at the level of insufficient evidence [4, 6]. Given these, we carried out this meta-analysis study of RCTs in a hope to summarize laparoscopic resection data comparing NOSE versus conventional laparoscopy in colorectal cancer.

## 2. Methods

### 2.1. Study Search

We identified eligible RCTs by searching seven electronic databases (PubMed, Cochrane Library, Embase, Web of Science, CNKI, CQVIP, Wanfang, and Sinomed) by using the following terms: “colorectal disease” or “colorectal cancer” or “colorectal tumor” or “colorectal carcinoma” or “colorectal neoplasm” or “rectal disease” or “rectal cancer” or “rectal tumor” or “rectal carcinoma” or “rectal neoplasm” and “natural orifice specimen extraction surgery” or “natural orifice transluminal extraction surgery” or “transrectal specimen extraction” or “transrectal specimen extraction” or “transvaginal specimen extraction” or “no auxiliary incision” or “without auxiliary incision” or “NOSES” or “natural orifice transluminal endoscopic surgery (NOTES)”. Additionally, the references of relevant studies on the same topic were manually searched further.

### 2.2. Study Selection

All studies were carefully assessed for their appropriateness using the study entry criteria as follows: (1) published as original article of RCTs, (2) reported a diagnosis of colorectal cancer as study disease and compared the laparoscopic resection with NOSE versus conventional laparoscopic surgery, and (3) the report language was Chinese or English. If more than one article reported data from the same study, the most recent and complete articles were included. However, those studies without any valid information on resection outcomes were removed.

### 2.3. Data Extraction and Quality Assessment

In this meta-analysis between laparoscopic resection with NOSE surgery (NOSE group) and conventional laparoscopy (CL group), the following data were extracted from each eligible individual study: (1) the name of first author; (2) year of publication; (3) study groups and number of patients; (4) baseline characteristics such as age and sex; and (5) resection outcomes including operation time, estimated blood loss, gas passage after surgery, various complications, and duration of hospital stay.

Two investigators utilized a uniform structured extraction sheet to extract data from included RCTs. If any disagreement was noted, a third investigator was asked to reach a final agreement. The potential risk of study bias was assessed according to the preferred reporting items for systematic reviews and meta-analysis recommendations [7]. The level of evidence was evaluated by using the Oxford Levels of Evidence [8, 9]. Study quality was assessed by using the modified Jadad scale, which involves six items to evaluate the methodological quality of RCTs [10–12]. Its score range was 0 to 8, with a higher score showing better report quality. In this study, a score of 1 to 3 indicated low quality and 4 to 8 for high quality.

### 2.4. Statistical Analysis

We used R 3.4.4 (R Foundation for Statistical Computing, Vienna, Austria; http://www.R-project.org/) and the Meta package [13] for this meta-analysis. For continuous outcome data, mean differences (MDs) along with their 95% confidence intervals (CIs) were used as their main effect measures. When the mean and standard deviation were not provided directly, we estimated them from the median, range, and size of the study samples [14]. For binary event data, the rate ratios (RRs) were calculated with 95% CIs. Heterogeneity was defined as an *I*^2^ value of more than 50% [15] or *p* value of less than 0.10 from Cochrane Q test [16]. These two statistics evaluate the percentage of variability attributable to study heterogeneity instead of by chance. Therefore, when an outcome measure showed negligible heterogeneity, we used a fixed-effect model for its data pooling instead of random-effects model. The funnel plots were visually inspected for the measures of most included RCTs being conducted to statistically evaluate publication bias [15]. For any statistical test, significance was defined as a two-tailed *p* value of 0.05 or less.

## 3. Results

### 3.1. Search Results and Study Characteristics

**Figure 1:**
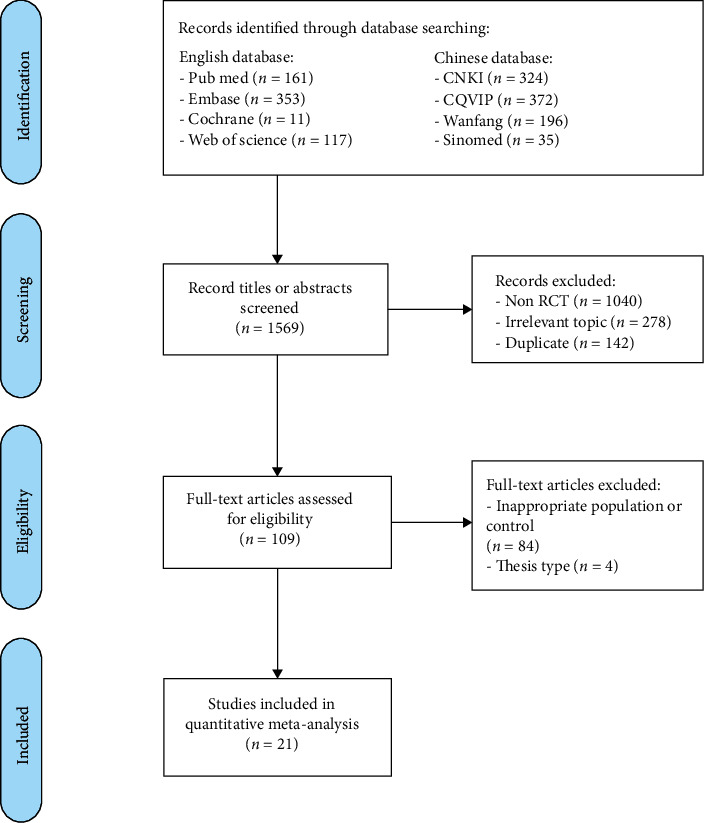
Flow diagram of the study search and selection process.

Initially, 1,569 citations were identified from electronic database as of June 2020 (cut-off date), of which 1,460 were excluded for a variety of reasons after the screening of citation titles and abstracts, leaving 109 studies for further full-text assessment. Of them, 88 studies were excluded due to their inappropriate study population or thesis type. Finally, a total of 21 RCTs [17–37] involving 2,112 patients met the study eligibility criteria and were included (Figure 1).

Only patients from the NOSE group or the CL group according to laparoscopic resection methods were included in our meta-analysis. Four studies [38–41] published as thesis and not in peer-reviewed journals were excluded. One study [42] with a printing error but was repaired and one study [43] in Russian were excluded. For four studies with more than two arms, we removed the open surgery group from two studies [19, 27] and the laparoscopic surgery plus a traditional nursing group [30] or combined two NOSES-type arms into one [32]. The main study characteristics are shown in Table 1.

### 3.2. Study Quality and Publication Bias

The results of quality assessment by the modified Jadad scale were as follows: two articles scored 6, five scored 5, twelve scored 4, one scored 3, and one scored 2. In summary, 19 out of 21 studies earned a score of 4 or more. All of the 21 articles were on RCT design and met 1b level of evidence. These generally suggested their high study quality (Table 2).

**Figure 2:**
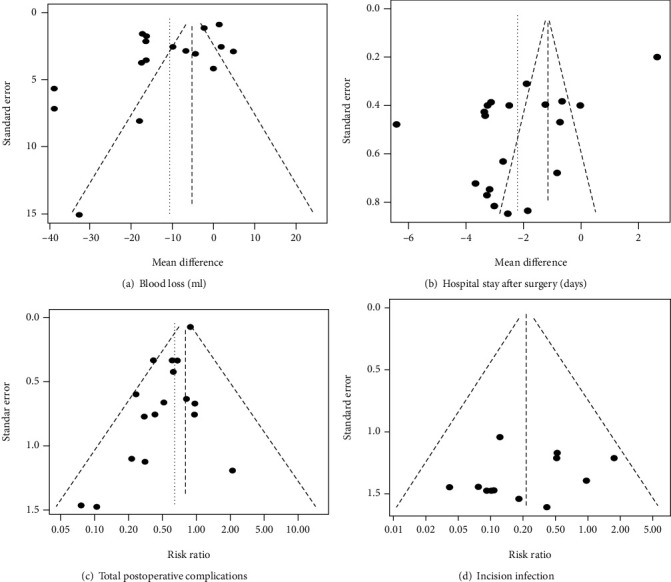
Funnel plot of the meta-analysis using the rate ratios against their standard errors. (a) Estimated blood loss in millilitres; (b) hospital stay after surgery in days; (c) total postoperative complications; (d) incision infection.

The funnel plots were drawn for effect outcomes of estimated blood loss, hospital stay after surgery, total postoperative complications, and incision infection (Figure 2). Incision infection showed some symmetry, and no statistically significant publication bias was found (*p* = 0.3103). Funnel plots for the other outcomes showed asymmetry (Figure 2).

### 3.3. Meta-Analysis Results

#### 3.3.1. Intraoperative Data and Postoperative Recovery

**Figure 3:**
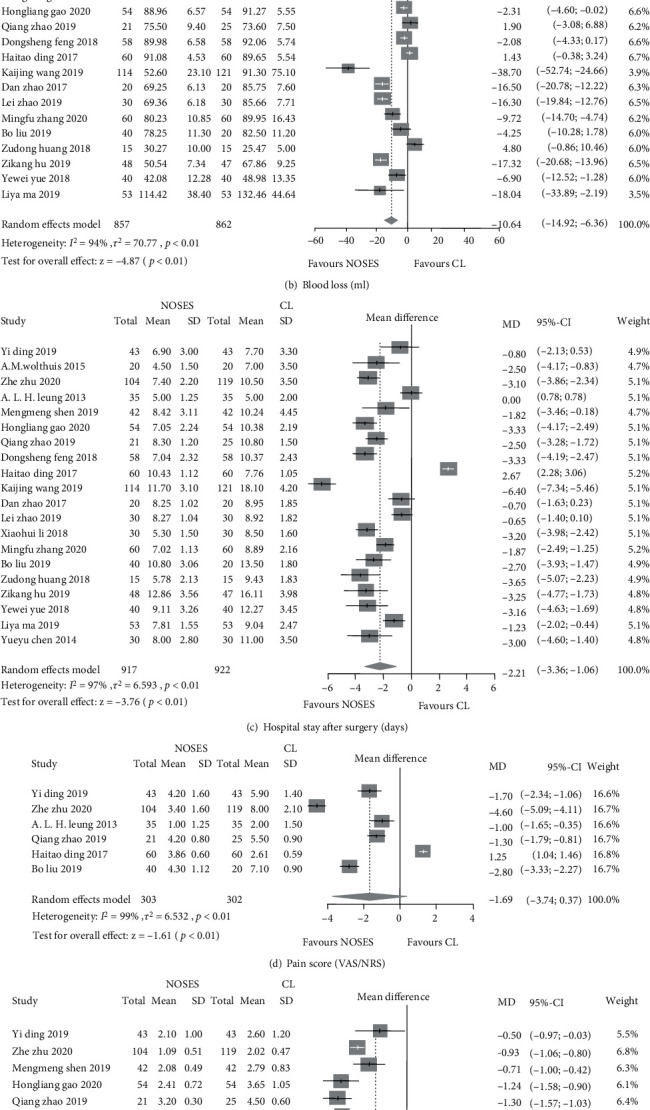
Forest plots of intraoperative data and postoperative recovery between the NOSE group and the CL group. (a) Operation time in minutes; (b) estimated blood loss in millilitres; (c) hospital stay after surgery in days; (d) pain score; (e) gas passage after surgery in days; (f) cosmetic result.

The patient intraoperative data and postoperative recovery of the included RCTs are presented in Table 3. Operation time, estimated blood loss, and hospital stay after surgery were reported in 18, 18, and 20 studies, respectively. An 8 minutes of mean operation time was prolonged in the NOSE group as compared to the CL group (MD: 8.14 min, 95% CI: 3.02 to 13.25, and *p* < 0.01). However, intraoperative estimated blood loss was decreased in the NOSE group as compared to the CL group (MD: -10.64 ml, 95% CI: -14.92 to -6.36, and *p* < 0.01). Moreover, hospital stay after surgery was shortened in the NOSE group significantly (MD: -2.21 days, 95% CI: -3.36 to -1.06, and *p* < 0.01). Gas passage after surgery was reported in 16 studies and was also shortened in the NOSE group (MD: -0.58 days, 95% CI: -0.82 to -0.34, and *p* < 0.01); pain score was improved in the NOSE group (MD: -1.06, 95% CI: -3.74 to -0.37, and *p* < 0.01); cosmetic result seemed better in the NOSE group (MD: 1.93, 95% CI: 0.77 to 3.10, and *p* < 0.01) (see Figure 3 for details).

#### 3.3.2. Postoperative Complications

**Figure 4:**
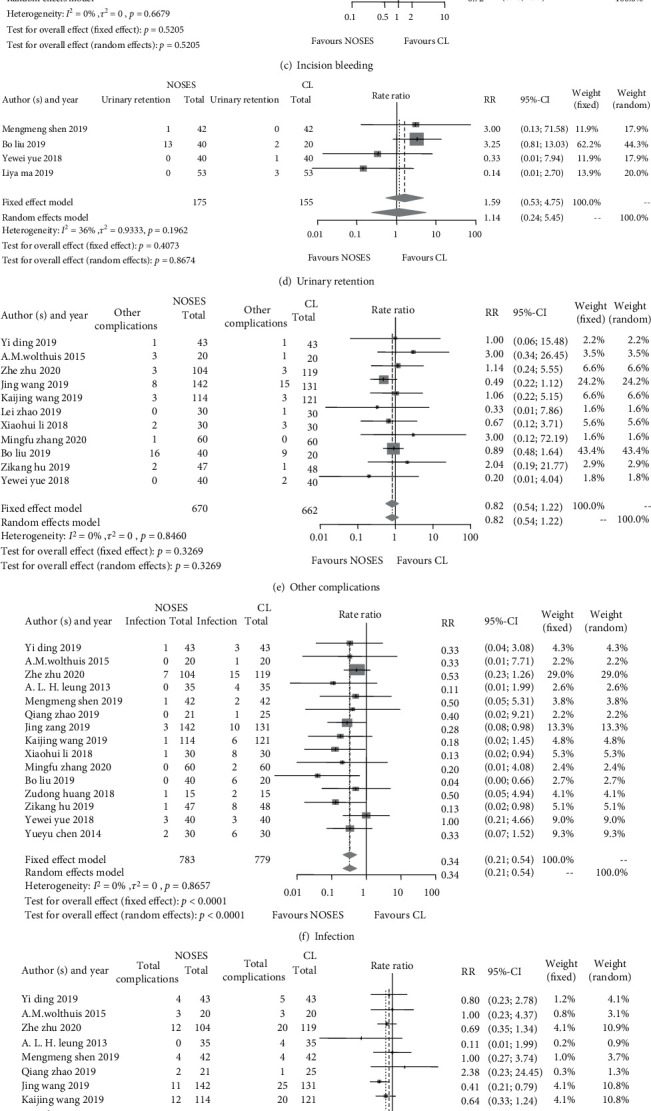
Forest plots of postoperative complications between the NOSE group and the CL group. (a) Anastomotic leakage; (b) ileus; (c) incision bleeding; (d) urinary retention; (e) other complications; (f) infection; (g) total complications; (h) incision infection.

The postoperative complications of the included RCTs are presented in Table 4, and various postoperative infections are detailed in Table 5. Postoperative complications were reported in 18 RCTs. 102 out of 886 patients (11.5%) developed postoperative complications in the NOSE group while 154 out of 882 patients (17.5%) in the CL group (RR of 0.81, 95% CI 0.71 to 0.93, and *p* = 0.003 in the fixed-effect model, Figure 4). And this improved trend was also shown in the postoperative infection (RR: 0.34, 95% CI: 0.21 to 0.54, and *p* < 0.0001), especially in the incision infection (RR: 0.24, 95% CI: 0.12 to 0.51, and *p* = 0.0002). However, no significant rate differences were found between the two groups in terms of anastomotic leakage (RR: 1.00, 95% CI: 0.53 to 1.90, and *p* = 0.9989), ileus (RR: 0.70, 95% CI: 0.19 to 2.60, and *p* = 0.5969), incision bleeding (RR: 0.72, 95% CI: 0.26 to 1.97, and *p* = 0.5205), urinary retention (RR: 1.14, 95% CI: 0.24 to 5.45, and *p* = 0.8674), and other complications (RR: 0.82, 95% CI: 0.54 to 1.22, and *p* = 0.3269) (Figure 4).

#### 3.3.3. Recurrence and Overall Survival

**Figure 5:**
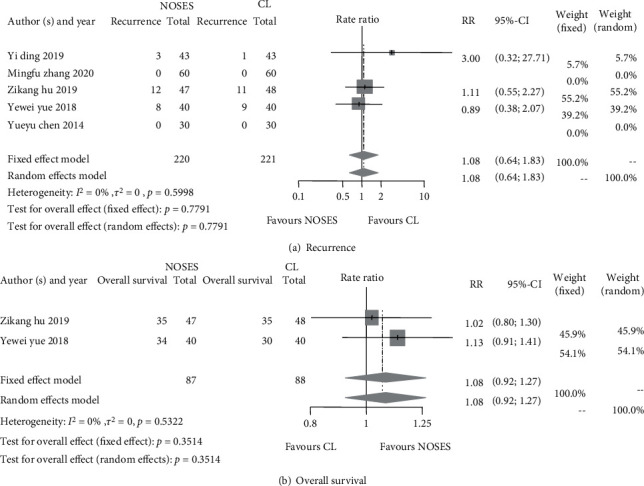
Forest plots of recurrence and overall survival rate between the NOSE group and the CL group. (a) Disease recurrence rate; (b) overall survival rate.

Disease recurrent data were reported in five studies and overall survival in two studies (Table 6). No significant differences for both survival-related outcomes were found between the two groups: RR of 1.08, 95% CI 0.64 to 1.83, and *p* = 0.7791 for event recurrence rate and 1.08, 95% CI 0.92 to 1.27, and *p* = 0.3514 for overall survival rate (Figure 5).

## 4. Discussion

To our knowledge, this report appeared the first comprehensive meta-analysis to synthesize RCT data regarding NOSE versus traditional laparoscopic colorectal cancer surgery. The large-sized meta-analysis of 21 RCTs demonstrated that laparoscopic resection with NOSE surgery reduced intraoperative estimated blood loss, relieved postoperative pain, accelerated postoperative recovery, and decreased the incidence of postoperative complications as well.

The terminology regarding NOSE surgery means that the surgical specimen resection is conducted intra-abdominally, and then, the specimen is taken out by opening a hollow organ such as anus, vagina, or mouth to communicate with the outside of the body [44]. Laparoscopic surgery combined with NOSE avoids incisions on the abdominal wall and reduces pain and wound complications, along with a shorter recovery time, etc. [45]. Besides, there was no auxiliary incision on the abdominal wall, and only a few small puncturing scars remained, indicating an excellent minimally invasive effect [46].

Given these reasons above, it was expected that NOSE surgery showed a better prognosis in terms of intraoperative data, postoperative recovery, and complications. NOSE surgery had less estimated blood loss (approximately 11 ml), and it may be due to no auxiliary incision, reducing the amount of wound bleeding. In the meantime, these results suggested that patients in NOSE group had less postoperative pain, faster recovery than the CL group, which also might be due to no auxiliary incision. The incidence of postoperative complications is an important indicator to evaluate the feasibility of NOSES. The total postoperative complication results suggested a significantly lower risk of complications (RR = 0.62, 95% CI 0.48 to 0.82, and *p* = 0.0006), especially in the incision infection. Therefore, in recent years, great advances in NOSES lead to a new tendency in CRC's surgical therapy in China and even other countries around the world. Given these, “Expert consensus of natural orifice specimen extraction surgery in colorectal neoplasm (2019)” and “International consensus on NOSES for colorectal cancer (2019)” were published along with individual reports [44, 46].

On the other hand, however, NOSE surgery had a slightly longer mean operation time (8 minutes) as compared to the CL group. The reasons behind it may include (1) the operation space inside the natural cavity is narrow so that the anastomosis is more time-consuming and (2) surgical proficiency of the surgeon with a possible learning curve. Beginners require a learning process to perform this new type of surgery. As for disease recurrence and overall survival rate, there was no significant difference noted between the NOSE group and the CL group, suggesting that there was likely no significant difference in the long-term efficacy. For postoperative complications, new studies with adequate sample size may be also needed to differentiate them later in the future. Even so, laparoscopic NOSES was, to some extent, a safe extraction method for colorectal diseases.

There were several limitations in this report. First, the meta-analysis was based on secondary study-level data, and the evaluation indicators varied greatly among different RCTs. Low quality of RCTs (2 out of 21 RCTs scored less than 4 by the modified Jadad scale) might influence the pooled results. Unlike one meta-analysis report recently published with only one RCT included [47], we only included RCTs (*n* = 21). Second, few studies reported the disease recurrence and overall survival data and the like. For them, it was difficult to adequately measure the long-term efficacy of NOSE surgery. Third, of the 21 included RCTs, one was reported in Belgium, one was in Hong Kong, China, and the others were all reported in mainland China. The enrolled studies were not widely distributed all over the world, which would limit the study finding to extrapolate further. Last, different operation skills and study population might induce potential bias among the included RCTs. Therefore, a large-sized well-controlled RCT is warranted to further verify the advantages and disadvantages of NOSES after following a uniform surgery guideline.

## 5. Conclusion

This report appeared the first comprehensive meta-analysis to quantitatively synthesize data from RCTs of laparoscopic resection with NOSE versus conventional laparoscopy. Compared with CL, NOSE surgery demonstrated multiple advantages in terms of shorter hospital stay after surgery, less pain, faster recovery from surgery, better cosmetic results, and most importantly, fewer postoperative complications. Even so, well-controlled RCTs of the NOSES following a uniform surgery guideline are warranted in the future.

## Figures and Tables

**Table 1:** Characteristic of 21 studies included in the meta-analysis.

No.	First author/year [Ref]	Country/region	Sample size	Age (years), mean ± SD or median (range)	Gender (*n*)		BMI (kg/m^2^), mean ± SD or median (range)
					Male	Female	
1.	Yi Ding/2019 [17]	China	43/43	56.48 ± 10.23/58.02 ± 9.66	25/22	18/21	23.6 ± 3.1/.2 ± 3.4
2.	A.M. Wolthuis/2015 [18]	Belgium	20/20	54 (31–72)/58 (40–73)	5/10	15/10	23.5 (18–29)/24 (20–29)
3.	Zhe Zhu/2020 [19]	China	104/119	61.4 ± 12.3/62.5 ± 12.1	50/65	64/56	23.2 ± 1.6/24.4 ± 3.7
4.	A. L. H. Leung/2013 [20]	Hong Kong, China	35/35	62 (51–86)/72 (49–84)	13/12	22/23	-
5.	Mengmeng Shen/2019 [21]	China	42/42	58.61 ± 4.44/58.46 ± 4.21	20/18	22/24	22.16 ± 0.51/22.14 ± 0.62
6.	Hongliang Gao/2020 [22]	China	54/54	60.67 ± 6.95/61.93 ± 7.07	33/31	21/23	-
7.	Qiang Zhao/2019 [23]	China	21/25	58.2 ± 7.6/50.5 ± 6.8	12/15	9/10	26.4 ± 7.9/27.3 ± 8.2
8.	Dongsheng Feng/2018 [24]	China	58/58	59.14 ± 5.97/59.09 ± 6.14	37/35	21/23	-
9.	Jin Wang/2019 [25]	China	142/131	60.9 ± 6.6/60.3 ± 6.4	75/72	67/59	-
10.	Haitao Ding/2017 [26]	China	60/60	58.33 ± 3.38/58.26 ± 3.36	37	23	22.88 ± 2.66/22.87 ± 2.65
11.	Kaijing Wang/2019 [27]	China	114/121	61.4 ± 12.3/62.5 ± 12.1	50	64	23.2 ± 1.6/24.4 ± 3.7
12.	Dan Zhao/2017 [28]	China	20/20	52.15 ± 3.50/53.05 ± 4.50	12/13	8/7	21.7/20.6
13.	Lei Zhao/2019 [29]	China	30/30	40.5 ± 3.5/43.5 ± 3.5	18/17	12/13	21.4/21.7
14.	Xiaohui Li/2018 [30]	China	30/30	53.8 ± 11.4/54.7 ± 12.2	16/16	14/14	-
15.	Mingfu Zhang/2020 [31]	China	60/60	58.32 ± 5.49/57.69 ± 5.12	36/33	24/27	23.01 ± 1.44/22.85 ± 1.21
16.	Bo Liu/2019 [32]	China	40/20	64.7 ± 7.6/62.8 ± 8.3	25/13	15/7	-
17.	Zudong Huang/2018 [33]	China	15/15	61.8 ± 8.6/62.9 ± 7.6	10/9	5/6	-
18.	Zikang Hu/2019 [34]	China	48/47	59.05 ± 9.98/58.87 ± 10.25	25/23	23/24	-
19.	Yewei Yue/2018 [35]	China	40/40	56.87 ± 10.31/58.17 ± 11.24	23/21	17/19	22.14 ± 1.87/21.79 ± 2.02
20.	Liya Ma/2019 [36]	China	53/53	54.72 ± 7.51/54.50 ± 7.32	28/27	25/26	23.17 ± 1.50/23.24 ± 1.35
21.	Yueyu Chen/2014 [37]	China	30/30	66.0 ± 1.4/67.0 ± 9.5	17/14	13/16	-

Note: Values are mean ± standard deviation (SD) or median (range) and “-” for not reported. Sample size, age, gender, and body mass index (BMI) data were supplied in the form of NOSES/CL separately.

**Table 2:** Level of evidence and modified Jadad quality score for the 21 included studies.

No.	First author/year [Ref]	Level of evidence^a^	Modified Jadad quality score with six items^b^						
			Randomization (2)	Blinding (2)	Withdrawals and dropouts (1)	Inclusion/exclusion criteria (1)	Adverse effects (1)	Statistical analysis (1)	Total
1.	Yi Ding/2019 [17]	1b	1	0	1	1	0	1	4
2.	A.M. Wolthuis/2015 [18]	1b	2	1	1	1	0	1	6
3.	Zhe Zhu/2020 [19]	1b	1	0	1	1	1	1	5
4.	A. L. H. Leung/2013 [20]	1b	1	1	1	1	1	1	6
5.	Mengmeng Shen/2019 [21]	1b	1	0	0	1	1	1	4
6.	Hongliang Gao/2020 [22]	1b	1	0	0	0	0	1	2
7.	Qiang Zhao/2019 [23]	1b	1	0	0	1	1	1	4
8.	Dongsheng Feng/2018 [24]	1b	1	0	0	0	1	1	3
9.	Jin Wang/2019 [25]	1b	1	0	0	1	1	1	4
10.	Haitao Ding/2017 [26]	1b	1	0	0	1	1	1	4
11.	Kaijing Wang/2019 [27]	1b	1	0	0	1	1	1	4
12.	Dan Zhao/2017 [28]	1b	1	0	1	1	1	1	5
13.	Lei Zhao/2019 [29]	1b	1	0	0	1	1	1	4
14.	Xiaohui Li/2018 [30]	1b	1	0	0	1	1	1	4
15.	Mingfu Zhang/2020 [31]	1b	1	0	0	1	1	1	4
16.	Bo Liu/2019 [32]	1b	1	0	0	1	1	1	4
17.	Zudong Huang/2018 [33]	1b	1	0	0	1	1	1	4
18.	Zikang Hu/2019 [34]	1b	1	0	1	1	1	1	5
19.	Yewei Yue/2018 [35]	1b	1	0	1	1	1	1	5
20.	Liya Ma/2019 [36]	1b	1	0	0	1	1	1	4
21.	Yueyu Chen/2014 [37]	1b	1	0	1	1	1	1	5

^a^Level of evidence was evaluated by using the Oxford Levels of Evidence (http://www.cebm.net/oxford-centre-evidence-based-medicine-levels-evidence-march-2009); ^b^the modified Jadad scale was composed of 6 items, including the following: (i) randomization (yes scored 2 points and no scored 0), (ii) blinding (yes scored 2 and no scored 0), (iii) description of withdrawals and dropouts (yes scored 1 point and no scored 0 points), (iv) inclusion/exc1usion criteria (yes scored 1 point and no scored 0 points), (v) adverse effects (yes scored 1 and no scored 0), and (vi) statistical analysis (yes scored 1 and no scored 0).

**Table 3:** Intraoperative data and postoperative recovery of studies included in the meta-analysis.

No.	First author/year [Ref]	*N*	Operation time (min)	Blood loss (ml)	Hospital stay after surgery (days)	Pain score (VAS/NRS)	Gas passage after surgery (days)	Cosmetic result
1.	Yi Ding/2019 [17]	43/43	131.59 ± 26.43/123.28 ± 23.87	59.31 ± 14.64/75.41 ± 18.16	6.9 ± 3.0/7.7 ± 3.3	4.2 ± 1.6/5.9 ± 1.4^a^	2.1 ± 1.0/2.6 ± 1.2	8.0 ± 1.5/6.4 ± 1.1
2.	A.M. Wolthuis/2015 [18]	20/20	90 (70–125)/75 (50–160)	10 (0–100)/0 (0–250)	4 (2–8)/4 (3–17)	3.5/2.1^a^	-	21 (14-24)/18 (8-24)
3.	Zhe Zhu/2020 [19]	104/119	166.2 ± 42.1/147.0 ± 45.0	52.6 ± 23.1/91.3 ± 56.7	7.4 ± 2.2/10.5 ± 3.5	3.4 ± 1.6/8 ± 2.1^a^	1.09 ± 0.51/2.02 ± 0.47	-
4.	A. L. H. Leung/2013 [20]	35/35	105 (60–170)/100 (59–210)	30 (10–50)/30 (10–100)	5 (4–9)/5 (3–11)	1 (0–5)/2 (0–6)^a^	-	-
5.	Mengmeng Shen/2019 [21]	42/42	182.61 ± 42.11/134.23 ± 28.71	72.45 ± 15.83/89.85 ± 18.51	8.42 ± 3.11/10.24 ± 4.45		2.08 ± 0.49/2.79 ± 0.83	-
6.	Hongliang Gao/2020 [22]	54/54	123.92 ± 6.58/125.74 ± 7.67	88.96 ± 6.57/91.27 ± 5.55	7.05 ± 2.24/10.38 ± 2.19		2.41 ± 0.72/3.65 ± 1.05	-
7.	Qiang Zhao/2019 [23]	21/25	140.6 ± 20.8/132.2 ± 16.2	75.5 ± 9.4/73.6 ± 7.5	8.3 ± 1.2/10.8 ± 1.5	4.2 ± 0.8/5.5 ± 0.9^b^	3.2 ± 0.3/4.5 ± 0.6	-
8.	Dongsheng Feng/2018 [24]	58/58	122.95 ± 6.95/126.97 ± 6.75	89.98 ± 6.58/92.06 ± 5.74	7.04 ± 2.32/10.37 ± 2.43	-	2.42 ± 0.75/3.64 ± 1.03	-
9.	Jin Wang/2019 [25]	142/131	-	-	-	-	-	-
10.	Haitao Ding/2017 [26]	60/60	124.06 ± 5.48/125.33 ± 5.54	91.08 ± 4.53/89.65 ± 5.54	10.43 ± 1.12/7.76 ± 1.05	3.86 ± 0.60/2.61 ± 0.59^b^	3.58 ± 0.61/2.54 ± 0.52	-
11.	Kaijing Wang/2019 [27]	114/121	167.0 ± 45.0/146.2 ± 42.1	52.6 ± 23.1/91.3 ± 75.1	11.7 ± 3.1/18.1 ± 4.2	-	0.67 ± 0.25/1.04 ± 0.26	-
12.	Dan Zhao/2017 [28]	20/20	180.6 ± 25.8/150 ± 14.4	69.25 ± 6.13/85.75 ± 7.60	8.25 ± 1.02/8.95 ± 1.85	-	-	-
13.	Lei Zhao/2019 [29]	30/30	187.2 ± 25.2/153 ± 14.4	69.36 ± 6.18/85.66 ± 7.71	8.27 ± 1.04/8.92 ± 1.82	-	-	-
14.	Xiaohui Li/2018 [30]	30/30	-	-	5.3 ± 1.5/8.5 ± 1.6	-	1.01 ± 0.14/1.50 ± 0.17	-
15.	Mingfu Zhang/2020 [31]	60/60	129.32 ± 15.21/125.04 ± 12.28	80.23 ± 10.85/89.95 ± 16.43	7.02 ± 1.13/8.89 ± 2.16	-	2.02 ± 0.51/2.89 ± 0.73	-
16.	Bo Liu/2019 [32]	40/20	186.4 ± 17.9/169.8 ± 18.3	78.25 ± 11.3/82.5 ± 11.2	10.8 ± 3.06/13.5 ± 1.8	4.3 ± 1.12/7.1 ± 0.9^b^	2.48 ± 0.64/2.35 ± 0.58	-
17.	Zudong Huang/2018 [33]	15/15	145.39 ± 39.61/123.94 ± 45.37	30.27 ± 10.00/25.47 ± 5.00	5.78 ± 2.13/9.43 ± 1.83	-	1.84 ± 0.78/1.76 ± 0.64	-
18.	Zikang Hu/2019 [34]	47/48	-	50.54 ± 7.34/67.86 ± 9.25	12.86 ± 3.56/16.11 ± 3.98	-	2.12 ± 1.04/3.49 ± 1.37	-
19.	Yewei Yue/2018 [35]	40/40	159.73 ± 21.49/150.18 ± 20.39	42.08 ± 12.28/48.98 ± 13.35	9.11 ± 3.26/12.27 ± 3.45	-	2.07 ± 0.53/2.68 ± 0.72	-
20.	Liya Ma/2019 [36]	53/53	184.72 ± 42.35/228.18 ± 45.03	114.42 ± 38.40/132.46 ± 44.64	7.81 ± 1.55/9.04 ± 2.47	-	3.01 ± 1.05/3.88 ± 1.26	-
21.	Yueyu Chen/2014 [37]	30/30	118.5 ± 22.0/138.1 ± 23.8	-	8.0 ± 2.8/11.0 ± 3.5	-	3.40 ± 0.23/3.59 ± 0.36	-

Note: Values are mean ± standard deviation (SD) or median (range); “-” for data not reported; data are supplied in the form of NOSES/CL separately. ^a^Pain score using VAS; ^b^pain score using NRS.

**Table 4:** Postoperative complications of studies included in the meta-analysis.

No.	First author/year [Ref]	*N*	Anastomotic leakage	Ileus	Incision bleeding	Urinary retention	Infection^a^	Other complications	Total
1.	Yi Ding/2019 [17]	43/43	1/0	1/1	-	-	1/3	1/1	4/5
2.	A.M. Wolthuis/2015 [18]	20/20	0/1	-	-	-	0/1	3/1	3/3
3.	Zhe Zhu/2020 [19]	104/119	2/2	-	-	-	7/15	3/3	12/20
4.	A. L. H. Leung/2013 [20]	35/35	0/0	-	-	-	0/4	-	0/4
5.	Mengmeng Shen/2019 [21]	42/42	1/1	1/1	-	1/0	1/2	-	4/4
6.	Hongliang Gao/2020 [22]	54/54	-	-	-	-	-	-	-
7.	Qiang Zhao/2019 [23]	21/25	1/0	1/0	-	-	0/1	-	2/1
8.	Dongsheng Feng/2018 [24]	58/58	-	-	-	-	-	-	-
9.	Jin Wang/2019 [25]	142/131	-	-	-	-	3/10	8/15	11/25
10.	Haitao Ding/2017 [26]	60/60	-	-	-	-	-	-	-
11.	Kaijing Wang/2019 [27]	114/121	8/11	-	-	-	1/6	3/3	12/20
12.	Dan Zhao/2017 [28]	20/20	0/0	-	0/0	-	-	-	0/0
13.	Lei Zhao/2019 [29]	30/30	-	-	1/2	-	-	0/1	1/3
14.	Xiaohui Li/2018 [30]	30/30	-	-	-	-	1/8	2/3	3/11
15.	Mingfu Zhang/2020 [31]	60/60	-	0/1	0/1	-	0/2	1/0	1/4
16.	Bo Liu/2019 [32]	40/20	6/2	-	-	13/2	0/6	16/9	35/19
17.	Zudong Huang/2018 [33]	15/15	-	-	1/3	-	1/2	-	2/5
18.	Zikang Hu/2019 [34]	47/48	-	-	4/3	-	1/8	2/1	7/12
19.	Yewei Yue/2018 [35]	40/40	-	-	-	0/1	3/3	0/2	3/6
20.	Liya Ma/2019 [36]	53/53	-	0/3	-	0/3	-	-	0/6
21.	Yueyu Chen/2014 [37]	30/30	-	-	-	-	2/6	-	2/6

Note: “-” indicated data not reported; data are supplied in the form of NOSES/CL separately; ^a^specific postoperative infection data are presented in Table 5.

**Table 5:** Postoperative infection of studies included in the meta-analysis.

No.	First author/year [Ref]	*N*	Incision infection	Pulmonary infection	Intraperitoneal infection	Urinary tract infection	Subtotal
1.	Yi Ding/2019 [17]	43/43	0/2	1/1	0/0	-	1/3
2.	A.M. Wolthuis/2015 [18]	20/20	-	-	-	0/1	0/1
3.	Zhe Zhu/2020 [19]	104/119	0/5	6/9	1/1	-	7/15
4.	A. L. H. Leung/2013 [20]	35/35	0/4	-	-	-	0/4
5.	Mengmeng Shen/2019 [21]	42/42	1/2	-	-	-	1/2
6.	Hongliang Gao/2020 [22]	54/54	-	-	-	-	-
7.	Qiang Zhao/2019 [23]	21/25	0/1	-	-	-	0/1
8.	Dongsheng Feng/2018 [24]	58/58	-	-	-	-	-
9.	Jin Wang/2019 [25]	142/131	-	2/7	-	1/3	3/10
10.	Haitao Ding/2017 [26]	60/60	-	-	-	-	-
11.	Kaijing Wang/2019 [27]	114/121	0/5	-	-	1/1	1/6
12.	Dan Zhao/2017 [28]	20/20	-	-	-	-	-
13.	Lei Zhao/2019 [29]	30/30	-	-	-	-	-
14.	Xiaohui Li/2018 [30]	30/30	0/6	0/1	-	1/1	1/8
15.	Mingfu Zhang/2020 [31]	60/60	0/2	-	-	-	0/2
16.	Bo Liu/2019 [32]	40/20	0/6	-	-	-	0/6
17.	Zudong Huang/2018 [33]	15/15	1/2	-	-	-	1/2
18.	Zikang Hu/2019 [34]	47/48	1/8	-	-	-	1/8
19.	Yewei Yue/2018 [35]	40/40	2/1	1/2	-	-	3/3
20.	Liya Ma/2019 [36]	53/53	-	-	-	-	-
21.	Yueyu Chen/2014 [37]	30/30	1/1	0/4	-	1/1	2/6

Note: “-” indicated data not reported; data are supplied in the form of NOSES/CL separately.

**Table 6:** Recurrence and overall survival of studies included in the meta-analysis.

No.	First author/year [Ref]	Patients, *n*	Duration of follow-up, months	Recurrence, *n*	Overall survival, *n*
1.	Yi Ding/2019 [17]	43/43	(12-45)/(12-45)	3/1	-
2.	Mingfu Zhang/2020 [31]	60/60	(12-24)/(12-24)	0	-
3.	Zikang Hu/2019 [34]	47/48	24/24	12/11	35/35
4.	Yewei Yue/2018 [35]	40/40	24/24	8/9	34/30
5.	Yueyu Chen/2019 [37]	30/30	28 (3-48)/28 (3-48)	0/0	-

Note: -: not reported. Data are supplied in the NOSES/CL form.

## Data Availability

The data supporting this meta-analysis are from previously reported studies and datasets, which have been cited.

## Abbreviations

CRC: Colorectal cancer
CI: Confidence interval
CL: Conventional laparoscopy
ERAS: Enhanced recovery after surgery
MD: Mean difference
NA: Not applicable
NOSE: Natural orifice specimen extraction
NOSES: Natural orifice specimen extraction surgery
NOTES: Natural orifice transluminal endoscopic surgery
PRISMA: Preferred reporting items for systematic reviews and meta-analyses
RCTs: Randomized controlled trials
RR: Rate ratio.
